# An Exploratory One Health Assessment of the Human–Animal–Environment Interface in a Swamp Buffalo–Wetland System: A Study in Thale Noi, Thailand

**DOI:** 10.3390/ani16203309

**Published:** 2026-10-21

**Authors:** Po Patchara Loblay, Supaporn Somrup, Uraiwan Tongkaemkaew

**Affiliations:** Faculty of Agriculture, Thaksin University, Phatthalung 93210, Thailand; 6929950007@tsu.ac.th (P.P.L.); supaporn.s@tsu.ac.th (S.S.)

**Keywords:** knowledge, attitude, and practice (KAP), swamp buffalo, One Health, Thale Noi wetland, zoonotic diseases

## Abstract

This study aimed to analyze the relationship between buffalo health and the health of participating swamp buffalo keepers under the One Health framework in the Thale Noi wetland, Phatthalung Province, Thailand. An exploratory cross-sectional survey was conducted among 25 participating swamp buffalo keepers using a structured questionnaire covering socio-demographic characteristics, buffalo production systems, and knowledge, attitudes, and practices (KAP) related to zoonotic disease prevention and One Health. Data were analyzed using descriptive statistics. The results showed that most respondents were male (68.0%), had completed primary education (68.0%), and were engaged primarily in agriculture (96.0%). The average age was 57 ± 12 years, with an average of 32 ± 14 years of buffalo-raising experience and 31 ± 41 buffaloes per household. Most buffaloes were raised in wetland grazing systems (92.0%), reflecting local livelihoods’ close dependence on the Thale Noi wetland ecosystem. Respondents demonstrated a moderate-to-high level of knowledge about zoonotic diseases and buffalo health, with an overall knowledge score of 76.4%. However, only 40.0% correctly understood the One Health concept. Attitudes toward disease prevention and integrated health management were highest (mean = 4.53 ± 0.66), while preventive practices were also high (mean = 4.07 ± 1.16). Practices such as consulting veterinarians and maintaining hygiene were widely adopted, although consistent use of personal protective equipment remained relatively low. These findings provide preliminary exploratory evidence of positive attitudes and the adoption of appropriate preventive practices but need greater awareness of the One Health concept. Strengthening integrated education, disease surveillance, and collaborative management among the livestock, public health, and environmental sectors will help sustainably improve the health of buffaloes, humans, and the wetland ecosystem.

## Simple Summary

Swamp buffaloes play an important role in supporting rural livelihoods and maintaining the wetland ecosystem in the Thale Noi Wetland, southern Thailand. Because buffaloes, swamp buffalo keepers, wildlife, and the environment interact closely, disease prevention requires a One Health approach that considers the health of humans, animals, and ecosystems together. This study assessed the knowledge, attitudes, and practices of 25 participating swamp buffalo keepers regarding One Health and disease prevention. The participating swamp buffalo keepers demonstrated good practical knowledge, positive attitudes, and generally appropriate preventive practices. However, only 40% of respondents were familiar with the One Health concept, indicating that many participating swamp buffalo keepers understand disease prevention through experience rather than through an integrated health perspective. Based on these findings, we propose a One Health framework that links participating swamp buffalo keepers’ health, buffalo health, environmental health, veterinary services, farm management, and disease surveillance. The results provide practical information to improve community-based disease surveillance, strengthen extension programs, and support the sustainable management of traditional swamp buffalo farming and the Thale Noi wetland ecosystem.

## 1. Introduction

Emerging infectious diseases continue to pose major threats to global public health, with more than 60% of known infectious diseases and approximately 75% of emerging pathogens originating from animals. Accelerating environmental changes, biodiversity loss, agricultural intensification, and increasing interactions among humans, livestock, wildlife, and ecosystems have further increased the likelihood of zoonotic disease emergence. These complex challenges cannot be effectively addressed through human or veterinary medicine alone, requiring integrated approaches that consider the interconnected health of humans, animals, and the environment [1,2,3]. In this study, One Health was operationally defined as an integrated, multisectoral, and transdisciplinary approach that seeks to sustainably balance and optimize the health of people, animals, and ecosystems [4,5,6]. The conceptual foundation of the study was guided by the One Health definition and framework developed by the One Health High-Level Expert Panel (OHHLEP), which emphasizes the interconnectedness of human, animal, and ecosystem health and the need for coordinated action across relevant sectors. Accordingly, the OHHLEP framework was used to guide the conceptualization and organization of the questionnaire domains, with particular emphasis on the interrelationships among buffalo keepers, swamp buffaloes, wildlife, and the wetland environment. This framework provided the basis for assessing farmers’ knowledge, attitudes, and practices (KAP) within the interconnected human–animal–environment interface.

The One Health framework has therefore emerged as a multidisciplinary strategy that integrates human health, animal health, and environmental management to strengthen disease prevention, surveillance, and sustainable ecosystem management. Rather than focusing on isolated disease events, One Health emphasizes understanding the ecological interactions that drive disease emergence and supports coordinated surveillance across environmental, veterinary, and public health sectors. Recent advances further highlight that effective surveillance systems should simultaneously incorporate environmental determinants, animal health indicators, and human behavioral factors to improve early detection and prevention of infectious disease [1,2].

Swamp buffaloes (*Bubalus bubalis*) play an essential role in sustaining rural livelihoods, traditional agriculture, and wetland ecosystems throughout Southeast Asia. In Thailand, free-ranging swamp buffalo production systems are closely linked to natural wetlands, where buffaloes interact continuously with farmers, domestic animals, migratory birds, wildlife, and aquatic environments. These ecological interactions contribute to ecosystem services and biodiversity conservation while simultaneously creating potential pathways for pathogen transmission across human–animal–environmental interfaces, making swamp buffalo production an important context for applying the One Health approach [7,8,9]. Because farmers are frontline stakeholders in disease prevention and livestock management, understanding their KAP is fundamental to strengthening zoonotic disease surveillance and biosecurity. Previous KAP studies among livestock and wildlife farmers in Bangladesh and Vietnam have shown that farmers may hold positive attitudes toward disease prevention while still exhibiting important gaps in disease knowledge, preventive behaviors, and biosecurity practices. These studies have also identified education, farming experience, production systems, and biosecurity training as significant determinants influencing KAP outcomes [10,11]. More recent evidence from other livestock-producing settings further indicates that disease awareness does not necessarily translate into appropriate preventive practices. For example, a recent brucellosis KAP study involving 506 cattle farmers and veterinary professionals reported discrepancies between disease awareness, reported preventive behaviors, personal protective equipment (PPE) use, and professional veterinary knowledge [12]. Similarly, a study involving 533 livestock owners found that high general awareness of lumpy skin disease (LSD) coexisted with limited understanding of transmission pathways and inconsistent preventive practices [11]. Together, these findings highlight the importance of assessing not only farmers’ knowledge and attitudes toward disease prevention but also whether these are reflected in their reported preventive and biosecurity practices. In the context of swamp buffalo production, such an assessment should consider both the social and ecological dimensions of livestock management. This integrated perspective is particularly relevant to free-ranging systems, where interactions among farmers, buffaloes, wildlife, and wetland environments may create complex challenges for disease prevention and biosecurity. Identifying knowledge gaps and barriers to adopting appropriate practices can therefore inform interventions compatible with farmers’ everyday activities and the ecological characteristics of free-ranging production systems [13,14].

Despite the growing body of One Health and livestock KAP research, important gaps remain in understanding how One Health can be applied to traditional free-ranging swamp buffalo production in tropical wetland ecosystems. Existing studies have largely focused on zoonotic disease awareness, disease surveillance, or livestock biosecurity in conventional agricultural settings, with comparatively limited attention to production systems in which buffalo health, farmer health, wildlife interaction, and wetland environmental conditions are closely interconnected. Recent KAP evidence has also demonstrated that high disease awareness or positive attitudes do not necessarily translate into appropriate preventive and biosecurity practices [11,12]. However, evidence on integrating human, animal, and environmental dimensions within swamp buffalo production systems remains limited. This gap is particularly evident in the Thale Noi Wetland, an internationally important Ramsar wetland in Thailand, where traditional free-ranging swamp buffalo production occurs alongside ecologically sensitive wetland habitats and local communities [2,8]. Therefore, a context-specific assessment is needed to characterize the production system and examine how farmers’ KAP relate to the interconnected human–animal–environment interface.

Accordingly, this study has three specific objectives: (1) to assess the knowledge, attitudes, and practices (KAP) of participating swamp buffalo keepers regarding buffalo health, human health, and environmental management within a One Health perspective; (2) to characterize the traditional free-ranging swamp buffalo production systems and their interactions with participating swamp buffalo keepers, wildlife, and the wetland environment; and (3) to develop a context-specific conceptual framework integrating the study findings with established One Health principles to illustrate the interconnected human–animal–environment dimensions of swamp buffalo production in the Thale Noi Wetland. The findings are intended to provide baseline evidence to strengthen community-based One Health approaches, support appropriate disease prevention and biosecurity practices, and promote sustainable management of swamp buffalo production and wetland ecosystems.

## 2. Materials and Methods

### 2.1. Study Setting Area

**Figure 1:**
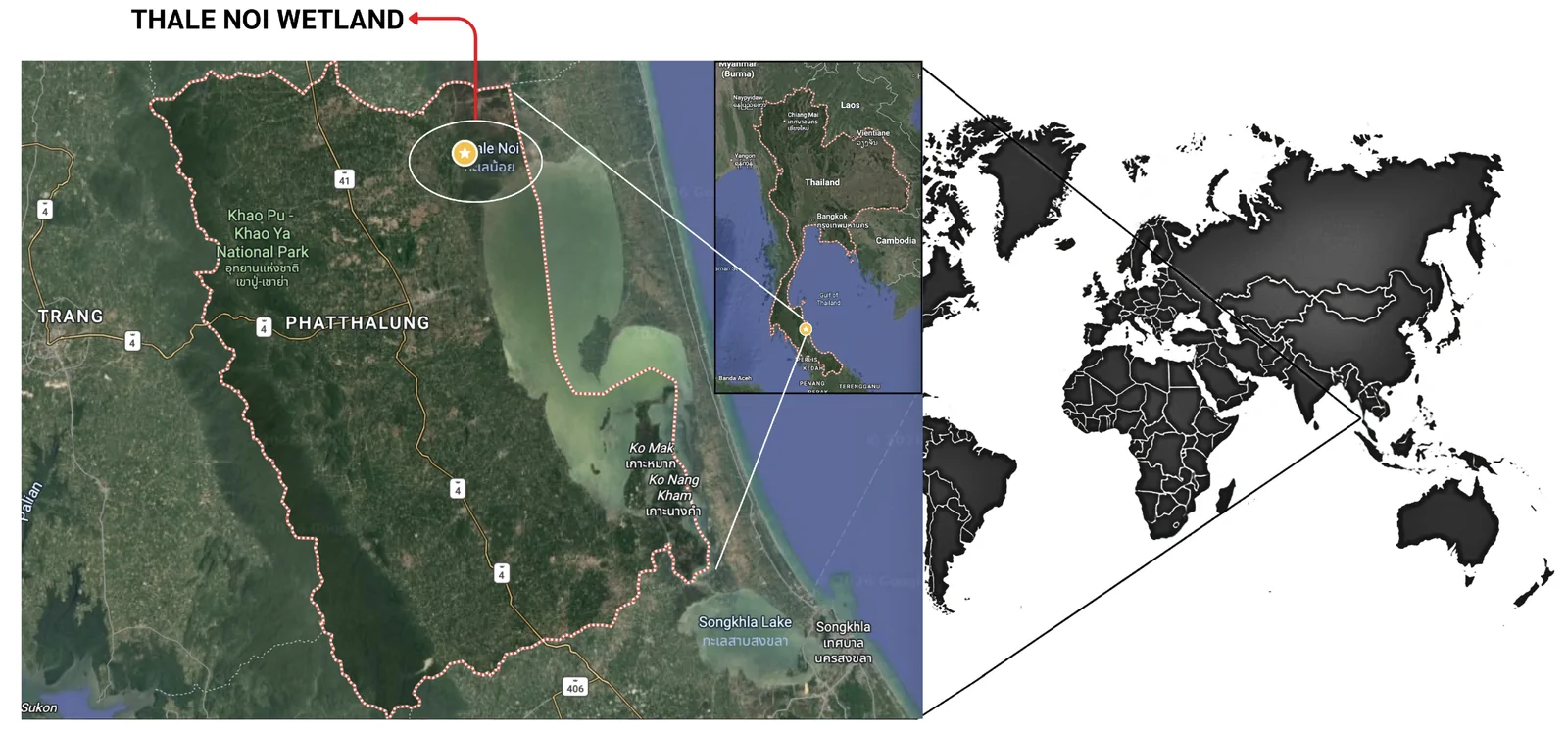
Location of the study area in the Thale Noi Wetland, Phatthalung Province, southern Thailand. The authors obtained base maps from Google Maps (© Google, 2026) and adapted them.

The study was conducted among participating swamp buffalo keepers raising swamp buffaloes in the Thale Noi Wetland, located in Khuan Khanun District, Phatthalung Province, southern Thailand (7°46′00″ N, 100°09′11″ E). Thale Noi is one of Thailand’s most important freshwater wetland ecosystems and is well known for its traditional free-ranging swamp buffalo production system, in which buffalo graze naturally within the wetland year-round (Figure 1). The area was designated Thailand’s first Non-Hunting Area in 1975, reflecting its outstanding ecological importance for conserving migratory waterbirds, native fish, and wetland biodiversity. The wetland provides a wide range of ecosystem services that support local livelihoods, including fishers, livestock grazing, ecotourism, and biodiversity conservation.

Thale Noi forms part of the Songkhla Lake basin, a unique lagoon ecosystem in southern Thailand. The broader cultural landscape, Songkhla and its Associated Lagoon Settlements, has been included in Thailand’s UNESCO Tentative List for World Heritage nomination, recognizing its outstanding interactions between humans and the wetland ecosystem over centuries. This recognition highlights the area’s ecological and cultural significance and reinforces the need to manage its natural resources sustainably. The coexistence of free-ranging swamp buffaloes, participating swamp buffalo keepers, wildlife, migratory birds, and aquatic ecosystems makes Thale Noi an ideal landscape for applying the One Health framework. Understanding interactions among animal health, human health, and environmental health is therefore essential to strengthen integrated disease surveillance, conserve biodiversity, and ensure the long-tserm sustainability of both the wetland ecosystem and the traditional swamp buffalo production system.

### 2.2. Study Design and Data Collection

The researchers conducted a cross-sectional survey between June and July 2026. The study was carried out in two sequential phases. In the first phase, the researchers conducted key informant interviews with officers from the Phatthalung Provincial Livestock Office and the Khaun Khanun District Livestock Office. The interviews aimed to obtain baseline information on the swamp buffalo-keeping population in the study area, including the geographical distribution of farming households, the approximate buffalo population, and livestock extension activities related to buffalo production, animal health management, and education for participating swamp buffalo keepers. Key informant information helped identify the study area, understand the local farming context, and support participant recruitment.

In the second phase, the study employed a purposive sampling approach and was designed as an exploratory study to obtain preliminary information on the practices, attitudes, and behaviors of participating swamp buffalo keepers who agreed to participate in the study. The findings from this phase were intended to describe the participating keepers and were not intended to be generalized to all swamp buffalo keepers in the study area. The target population comprised 122 swamp buffalo keepers identified in the study area, with an estimated total buffalo population of approximately 1662 animals, based on information obtained from the head-of-swamp buffalo keeper-group interview (2026). Initially, 62 swamp buffalo keepers were identified and approached for participation based on the predefined inclusion criteria and their relevance to the study objectives. Of these, 25 swamp buffalo keepers agreed to participate and provided complete information, while 37 did not participate in the study. Participation was voluntary, and swamp buffalo keepers who declined to participate were not replaced through random sampling. Eligible participants were swamp buffalo keepers actively engaged in buffalo keeping. No formal sample size calculation was performed before data collection because the study was an exploratory assessment rather than a population-based survey designed to generate a statistically representative sample of all 122 swamp buffalo keepers in the target population.

Data were collected through face-to-face structured questionnaires using a questionnaire consisting of eight sections: (1) general information; (2) knowledge, attitude, and practice (KAP); (3) human health; (4) environmental perception; (5) farm management; (6) buffalo health and disease management; (7) information utilization; and (8) recommendations. The KAP component comprised 10 knowledge items, 7 attitude items, and 8 practice items. The research team developed the questionnaire based on relevant literature on livestock-farmer KAP, zoonotic disease prevention, buffalo health management, and the One Health approach, rather than directly adopting a single previously validated questionnaire. The questionnaire assessed the interrelationship among human health, buffalo health, and environmental factors within the One Health framework.

Before the main survey, three experts with relevant expertise in livestock production and One Health reviewed the questionnaire to assess the relevance, clarity, and appropriateness of the items. Content validity was assessed using the Item-Objective Congruence (IOC) index. The IOC values ranged from 0.67 to 1.00, indicating acceptable content validity. The questionnaire was then pilot-tested with 11 swamp buffalo keepers not included in the main study sample but who had experience raising buffalo. The pilot test assessed the questionnaire’s clarity, comprehensibility, and appropriateness and identified ambiguous wording before the main data collection. Because the questionnaire was developed directly in Thai for Thai swamp buffalo keepers, translation and back-translation were not applicable. Before participation, all eligible swamp buffalo keepers were informed about the study’s purpose and procedures, and each participant provided written informed consent.

### 2.3. Data Analysis

Descriptive statistics were used to summarize the data, including frequency, percentage, mean, standard deviation, minimum, and maximum values. For variables with highly dispersed distributions, particularly herd size, we also reported the median and interquartile range (IQR) to provide a more robust description. Because the sample was limited and included only swamp buffalo keepers who agreed to participate, the findings related to the participating swamp buffalo keepers cannot be generalized to all swamp buffalo keepers in the Thale Noi area or elsewhere in Thailand. Instead, these findings describe only the specific group of swamp buffalo keepers enrolled in this exploratory study.

We calculated KAP scores separately for the knowledge, attitude, and practice components. For the knowledge component, each of the 10 items had three response options: “yes,” “no,” and “do not know.” Each response was scored against the predefined correct answer for the respective item. A correct response was assigned 1 point, whereas an incorrect response or “do not know” response was assigned 0 points. The total knowledge score therefore ranged from 0 to 10 points and was converted to a percentage by dividing the total score by 10 and multiplying by 100. The attitude component comprised 7 items, each rated on a 5-point Likert scale (1 = strongly disagree to 5 = strongly agree), resulting in a total possible score of 35 points. The practice component comprised 8 items, each rated on a 5-point frequency scale (1 = never to 5 = every time), resulting in a total possible score of 40 points. For the attitude and practice components, mean scores were categorized into five levels: Lowest (1.00–1.80), Low (1.81–2.60), Moderate (2.61–3.40), High (3.41–4.20), and Highest (4.21–5.00). The cut-off intervals were determined by dividing the total range of the 5-point scale (5 − 1 = 4) into five equal intervals of 0.80 points.

The descriptive findings were further qualitatively synthesized to identify potential interactions and health risks across the human, animal, and environmental domains among the study participants. This synthesis informed the development of the risk assessment under the One Health framework and the proposed One Health Conceptual Framework, which were presented and discussed in the Section 4.

## 3. Results

### 3.1. Participating Swamp Buffalo Keeper Characteristics

The socio-demographic characteristics of the participating swamp buffalo keepers are presented in Table 1 and Table 2. The respondents were predominantly male (17/25, 68.0%), while females accounted for 8/25 (32.0%) of the study population. Most participating swamp buffalo keepers had completed primary education (17/25, 68.0%), followed by secondary education (5/25, 20.0%) and vocational education (3/25, 12.0%). Agriculture was the principal occupation of 24/25 (96.0%) respondents, while 1/25 (4.0%) reported trading as the primary occupation. In addition to buffalo farming, 3/25 (12.0%) respondents reported having secondary occupations, indicating that a small proportion of participants engaged in additional occupation activities. The quantitative characteristics showed a mean age of 57 ± 12 years, while the mean farming experience was 32 ± 14 years. The average household size was 4 ± 1 members, and the mean herd size was 31 ± 41 swamp buffaloes, with substantial variation among households (range: 2–200 head). Annual income from swamp buffalo farming averaged 26,780 ± 9411.96 THB, ranging from 0 to 40,000 THB per year.

### 3.2. Buffalo Farming System

The swamp buffalo production system in the Thale Noi Wetland was characterized by long farming experience, variation in herd size, and predominant use of wetland areas for grazing (Table 3). Most participating swamp buffalo keepers (19/25, 76.0%) had more than 20 years of experience in buffalo husbandry, with an average farming experience of 32 ± 14 years, indicating that swamp buffalo production remains a long-established livelihood supported by substantial local knowledge and traditional management practices. Most farms maintained (9/25, 36%) herds of 21–50 buffaloes, followed by 7/25 (28.0%) who kept fewer than 10 animals, 5/25 (20.0%) who kept 10–20 animals, and 4/25 (16.0%) who kept more than 50 buffaloes.

Regarding production systems, 12/25 (48.0%) of participating swamp buffalo keepers reported using free-grazing systems and 12/25 (48.0%) reported using semi-intensive systems, while 1/25 (4.0%) reported another type of production system. For this study, free grazing was operationally defined as a production system in which buffaloes were primarily allowed to graze freely in the available grazing area, whereas semi-intensive production involved a combination of grazing and participating swamp buffalo keepers-managed feeding or confinement. The other category included production systems that did not fit these two predefined categories. Furthermore, 23/25 (92.0%) of participating swamp buffalo keepers identified wetlands as their primary grazing area, whereas 2/25 (8.0%) reported grasslands as their primary grazing area. These findings describe the production and grazing practices reported by participating swamp buffalo keepers in the study area and should not be generalized to all swamp buffalo keepers in the study area.

### 3.3. Knowledge

**Figure 2:**
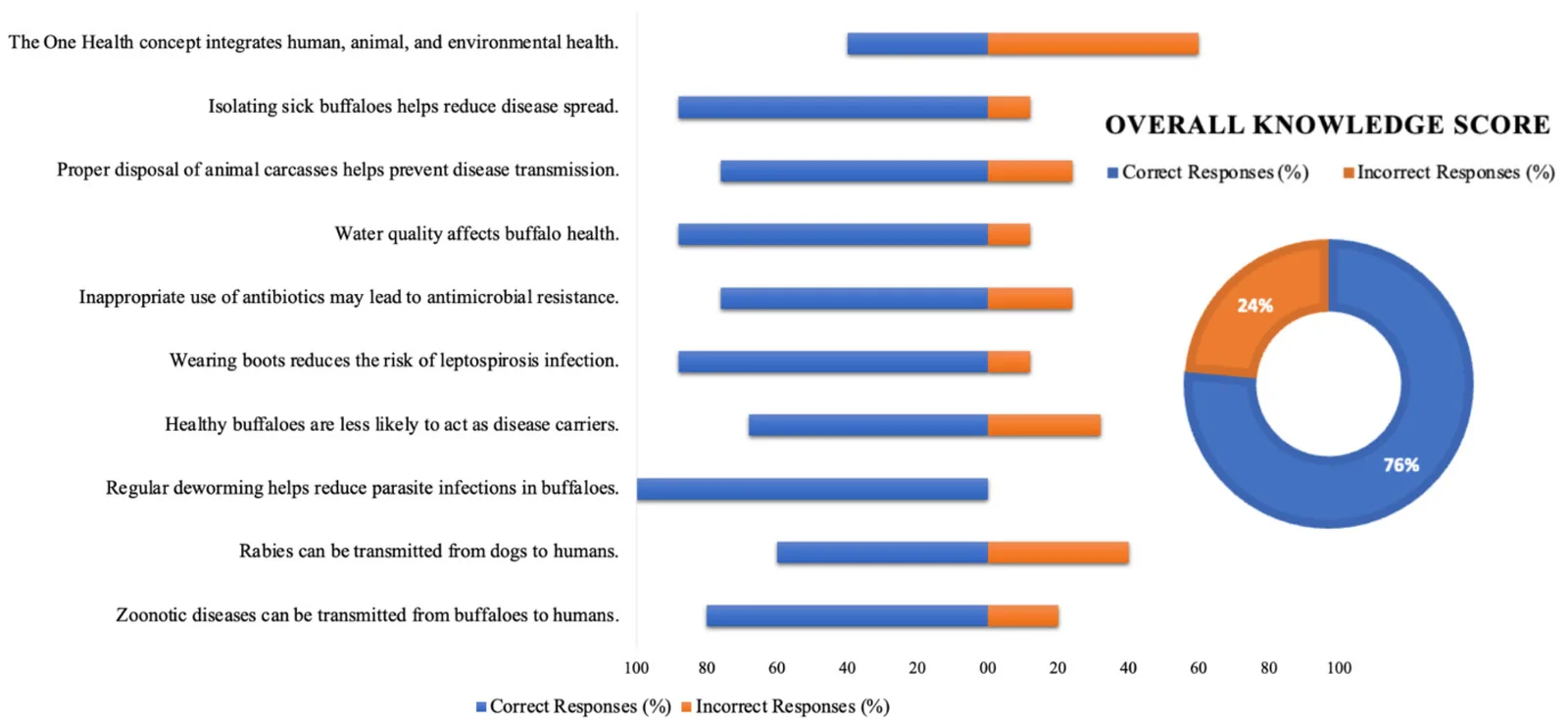
Knowledge of participating swamp buffalo keepers regarding human, buffalo, and environmental health under the One Health framework.

The knowledge assessment included 10 items covering human health, buffalo health, disease prevention, environmental health, and the One Health concept. Each item used three response options: “yes,” “no,” and “do not know.” Overall, the mean correct response rate was 76.4% (Figure 2). The highest proportion of correct responses was observed for proper waste disposal, with all 25 participating swamp buffalo keepers (100.0%) providing the correct response. Correct responses were also high for proper handwashing to reduce the risk of zoonotic diseases (22/25, 88%), good nutrition for buffalo health (22/25, 88.0%), and separation of sick buffaloes to reduce disease transmission (22/25, 88.0%). In addition, 20/25 (80.0%) correctly recognized that zoonotic disease can be transmitted from buffaloes to humans, while 19/25 (76.0%) correctly responded to the items concerning inappropriate antibiotic use and the role of accurate and timely animal health information in reducing disease transmission. Fewer correct responses were observed for the statement on the relative health status of strong and healthy buffaloes (17/25, 68.0%) and vaccination as a measure to prevent infectious disease in buffaloes (15/25, 60.0%). The lowest proportion of correct responses was observed for the One Health concept, with 10/25 participating swamp buffalo keepers (40.0%) correctly recognizing that One Health integrates human, animal, and environmental health. These findings indicate variation in participating swamp buffalo keepers’ knowledge across the individual One Health-related topics assessed, with relatively higher proportions of correct responses for practical disease-prevention and animal-health measures and a lower proportion for the broader One Health concept.

### 3.4. Attitude

The attitudes of participating swamp buffalo keepers toward health management under the One Health framework are presented in Table 4. The respondents demonstrated a highly positive attitude toward health management, with an overall mean score of 4.53 ± 0.66, corresponding to the highest attitude level. Respondents showed the strongest agreement that regular health check-ups for buffaloes are essential (mean = 4.72 ± 0.54), indicating that participating swamp buffalo keepers highly value routine animal health monitoring as a fundamental component of buffalo husbandry. Similarly, respondents expressed very positive attitudes toward community participation in disease surveillance and prevention (4.58 ± 0.65), disease prevention rather than treatment (4.56 ± 0.65), appropriate use of veterinary drugs according to veterinarians’ recommendations (4.56 ± 0.58), and the recognition that environmental quality directly influences the health of both buffaloes and humans (4.56 ± 0.71). Participating swamp buffalo keepers also strongly agreed that livestock officers or veterinarians should be notified when sick buffaloes are detected (4.52 ± 0.71). Among the assessed statements, the lowest attitude score, although still classified as high, was related to the routine use of personal protective equipment (PPE) when handling buffaloes (4.20 ± 0.82). This finding indicates comparatively lower acceptance of personal protective measures than other aspects of disease prevention and health management.

### 3.5. Practices Toward One Health-Based Disease Prevention and Buffalo Health Management

The practices of participating swamp buffalo keepers regarding One Health-based disease prevention and buffalo health management are presented in Table 5. The respondents demonstrated a high level of preventive practices, with an overall mean score of 4.07 ± 1.16, indicating that recommended disease prevention and buffalo health management practices were generally well adopted among the surveyed participating swamp buffalo keepers. The highest practice score was observed for seeking advice from a veterinarian when buffaloes became sick (4.28 ± 0.74), reflecting strong confidence in veterinary services and a willingness to seek professional support for animal health management. Similarly, participating swamp buffalo keepers frequently reported avoiding direct contact with buffalo blood or body fluids (4.20 ± 1.22), washing their hands after handling buffaloes or animal carcasses (4.16 ± 0.94), isolating sick buffaloes from the herd (4.12 ± 1.33), and regularly cleaning buffalo housing and equipment (4.12 ± 1.13). These findings indicate that basic biosecurity and hygiene measures are commonly practiced within the study area. In contrast, practice scores were comparatively lower for wearing boots when entering the buffalo grazing area (3.60 ± 1.32), routine deworming according to the recommended schedule (3.72 ± 1.28), and maintaining records of buffalo diseases, treatments, and drug use (3.92 ± 1.50). Although these practices remained in the high category, participating swamp buffalo keepers implemented them less consistently than other preventive measures.

## 4. Discussion

### 4.1. Participating Swamp Buffalo Keepers’ Health

The present study demonstrates that swamp buffalo farming in the Thale Noi Wetland is sustained by a community of participating swamp buffalo keepers with extensive farming experience, with respondents reporting an average of 32 years of buffalo farming experience. The participating swamp buffalo keepers showed a relatively high overall knowledge score (76.4%), highly positive attitudes (4.53 ± 0.66), and generally favorable practices (4.07 ± 1.16). However, these findings should not be interpreted as evidence that long-term farming experience directly contributed to higher KAP scores, as the present study did not statistically examine this relationship. Despite the relatively favorable overall KAP findings, important gaps remained in specific areas of knowledge and preventive practices; for example, only 40% of respondents correctly recognized the One Health concept, while the reported use of personal protective equipment (PPE) was less consistent than other preventive measures. These findings indicate that favorable overall KAP scores do not necessarily imply comprehensive understanding or consistent adoption of all recommended preventive measures. Similar disparities between general disease-prevention knowledge and specific preventive practices have also been reported in livestock production systems in Southeast Asia [10,11]. Among the participating swamp buffalo keepers, these findings indicate that favorable overall KAP scores do not necessarily imply comprehensive understanding or consistent adoption of all recommended preventive measures.

This pattern is consistent with recent evidence from livestock KAP research. Tursunkulov et al. [12] reported important gaps between farmers’ willingness to adopt preventive measures and their actual reported practices related to brucellosis transmission. Although farmers demonstrated awareness of disease prevention, the use of PPE and other protective measures during potentially high-risk activities remained limited. The study also found that animal health professionals had substantially higher knowledge than farmers [14]. These findings provide a useful comparison with the present study, in which participating swamp buffalo keepers also demonstrated positive attitudes toward disease prevention and veterinary consultation, while PPE-related practices were comparatively less consistent.

The relatively low proportion of participating swamp buffalo keepers who correctly identified the broader One Health concept further indicates an opportunity to strengthen integrated health education. Previous One Health research has highlighted similar concerns about limited understanding of the interconnectedness of human, animal, and environmental health [15]. Therefore, One Health interventions should not focus solely on increasing knowledge or maintaining positive attitudes but should also address practical barriers to preventive behaviors, including the accessibility, acceptability, and routine use of appropriate PPE and other biosecurity measures. Participatory learning, community engagement, and locally appropriate risk communication may help strengthen One Health literacy and support more consistent preventive practices among participating swamp buffalo keepers.

### 4.2. Buffalo Health

The buffalo production system observed in this study remains predominantly extensive, with almost all buffaloes relying on natural wetland grazing. This production system reflects the long-established relationship between swamp buffaloes and the Thale Noi ecosystem, where natural wetlands provide forage, water, and suitable habitats year-round (Table 6). Consequently, environmental conditions strongly influence buffalo health rather than farm management alone. Among the swamp buffalo keepers who participated in the study, awareness of routine herd health management was high, including parasite control, disease isolation, and consultation with veterinarians. However, practices requiring continuous management, such as scheduled deworming, health record-keeping, and routine biosecurity, were implemented less consistently. Similar management constraints have been reported in traditional buffalo production systems, where preventive measures requiring long-term planning are adopted less often than immediate husbandry practices [11,16]. These findings suggest that improving buffalo health among the participating swamp buffalo keepers should extend beyond treatment-oriented interventions toward preventive herd health management. Community-based disease reporting, systematic health recording, and regular veterinary monitoring would facilitate earlier detection of infectious disease while strengthening surveillance capacity in extensive production systems. These approaches align with One Health recommendations that emphasize integrated livestock health surveillance as a key component of sustainable disease prevention [17].

### 4.3. Environmental Health

Environmental health emerged as a central component of the traditional swamp buffalo production system in the Thale Noi Wetland. More than 90% of participating swamp buffalo keepers relied on wetlands as their primary grazing area, showing buffalo production’s close ecological dependence on ecosystem quality (Table 6). Wetland conditions influence forage availability, water quality, parasite ecology, and wildlife movement, thereby affecting both buffalo productivity and participating swamp buffalo keepers’ livelihoods. The extensive grazing system also creates continuous interactions among buffaloes, humans, wildlife, migratory birds, and aquatic ecosystems. Although this study did not directly investigate pathogen occurrence, these interactions represent potential pathways for disease transmission that warrant integrated surveillance. Contemporary One Health frameworks emphasize that environmental monitoring should complement animal and human health surveillance because ecological change often precedes the emergence or spread of infectious disease [2]. Therefore, strengthening environmental monitoring alongside veterinary and public health surveillance is particularly important for the Thale Noi Wetland. Coordinated collaboration among livestock authorities, environmental agencies, public health organizations, and local communities would support both biodiversity conservation and disease prevention. Such integrated governance is increasingly recognized as an essential strategy for sustaining livestock production in ecologically sensitive landscapes while improving resilience to emerging health and environmental challenges [14,18].

### 4.4. One Health Implications of the Study Findings

Based on this study’s findings, several potential health concerns and One Health implications were identified across the human, animal, and environmental domains. Although participating swamp buffalo keepers demonstrated relatively high knowledge, positive attitudes, and generally favorable reported practices, important gaps remained in One Health awareness and selected preventive practices. In addition, the dependence on wetland grazing and close interactions among buffaloes, humans, wildlife, and the aquatic environment highlight potential exposure pathways that warrant attention. These findings should be interpreted as potential health concerns identified from descriptive evidence rather than as results of a formal risk assessment, because the present study did not quantify the likelihood, exposure, or consequences of specific hazards or perform risk ranking; the findings therefore highlight areas that may benefit from integrated disease surveillance and cross-sector collaboration under the One Health framework.

#### 4.4.1. Participating Swamp Buffalo Keepers’ Health Implications

The present study identified limited One Health literacy as a key concern for participating swamp buffalo keepers’ health. Although respondents demonstrated strong practical knowledge of buffalo husbandry, only 40% correctly understood the One Health concept. This finding suggests that participating swamp buffalo keepers may recognize individual disease prevention measures without fully appreciating the interconnected relationships among human, animal, and environmental health. Similar knowledge gaps have been reported among livestock farmers in Southeast Asia, where practical husbandry knowledge frequently exceeds conceptual understanding of One Health and zoonotic disease prevention [11,19].

The comparatively lower acceptance of PPE-related practices also indicates an area for improvement in occupational biosecurity. Farmers routinely handle buffaloes in wetland environments where frequent contact with animals, water, and organic materials may increase exposure to zoonotic hazards. Previous studies similarly emphasize that strengthening personal biosecurity is an important component of community-based One Health programs [14,20]. Accordingly, extension activities should focus not only on disease awareness but also on practical behavioral support that facilitates the routine use of appropriate PPE during buffalo handling and other potentially high-exposure activities.

#### 4.4.2. Buffalo Health Implications

The buffalo production characteristics observed in this study indicate that buffalo health is closely linked to the wetland production environment. More than 90% of buffaloes grazed within wetlands, while free grazing and semi-intensive systems accounted for almost all production systems. These findings suggest frequent use of shared grazing areas and aquatic environments, where livestock, wildlife, and environmental components may interact. Similar ecological interfaces have been recognized as important drivers of infectious disease emergence within One Health surveillance systems [2]. Although farmers demonstrated good knowledge of disease prevention, they implemented several preventive practices—including scheduled deworming, herd health recording, and routine biosecurity—less consistently than veterinary consultation or hygiene measures. These reported practices indicate areas where herd health management could be strengthened, rather than increasing the likelihood of disease occurrence. Regular veterinary monitoring, community-based disease reporting, and systematic record-keeping may support earlier recognition of animal health problems and strengthen herd health management in traditional swamp buffalo production systems [11,17].

#### 4.4.3. Environmental Health Implications

Environmental health represents an important component of the traditional swamp buffalo production system in the Thale Noi Wetland. The overwhelming reliance on wetlands as grazing areas demonstrates that buffalo production is closely connected to the wetland ecosystem. Wetland conditions influence forage availability, water resources, and habitat connectivity, all of which affect buffalo production and the livelihoods of participating swamp buffalo keepers. The coexistence of buffaloes, participating swamp buffalo keepers, fisheries, migratory birds, wildlife, and aquatic ecosystems creates ecological interfaces through which interactions among different health domains may occur. Although this study did not directly investigate pathogen occurrence and transmission, the production characteristics identified here highlight the relevance of environmental monitoring as part of an integrated One Health surveillance approach. Contemporary One Health frameworks similarly emphasize that environmental surveillance complements veterinary and public health monitoring by identifying ecological changes that may precede disease emergence [2]. Furthermore, only 19/25 (76.0%) of respondents correctly recognized the importance of proper carcass disposal for disease prevention, indicating a knowledge gap in an environmental health-related preventive measure. Improving carcass management, environmental sanitation, and wetland conservation through collaboration among livestock authorities, environmental agencies, public health organizations, and local communities may contribute to reducing potential environmental health concerns and supporting the resilience of the wetland production system.

### 4.5. Proposed One Health Conceptual Framework

**Figure 3:**
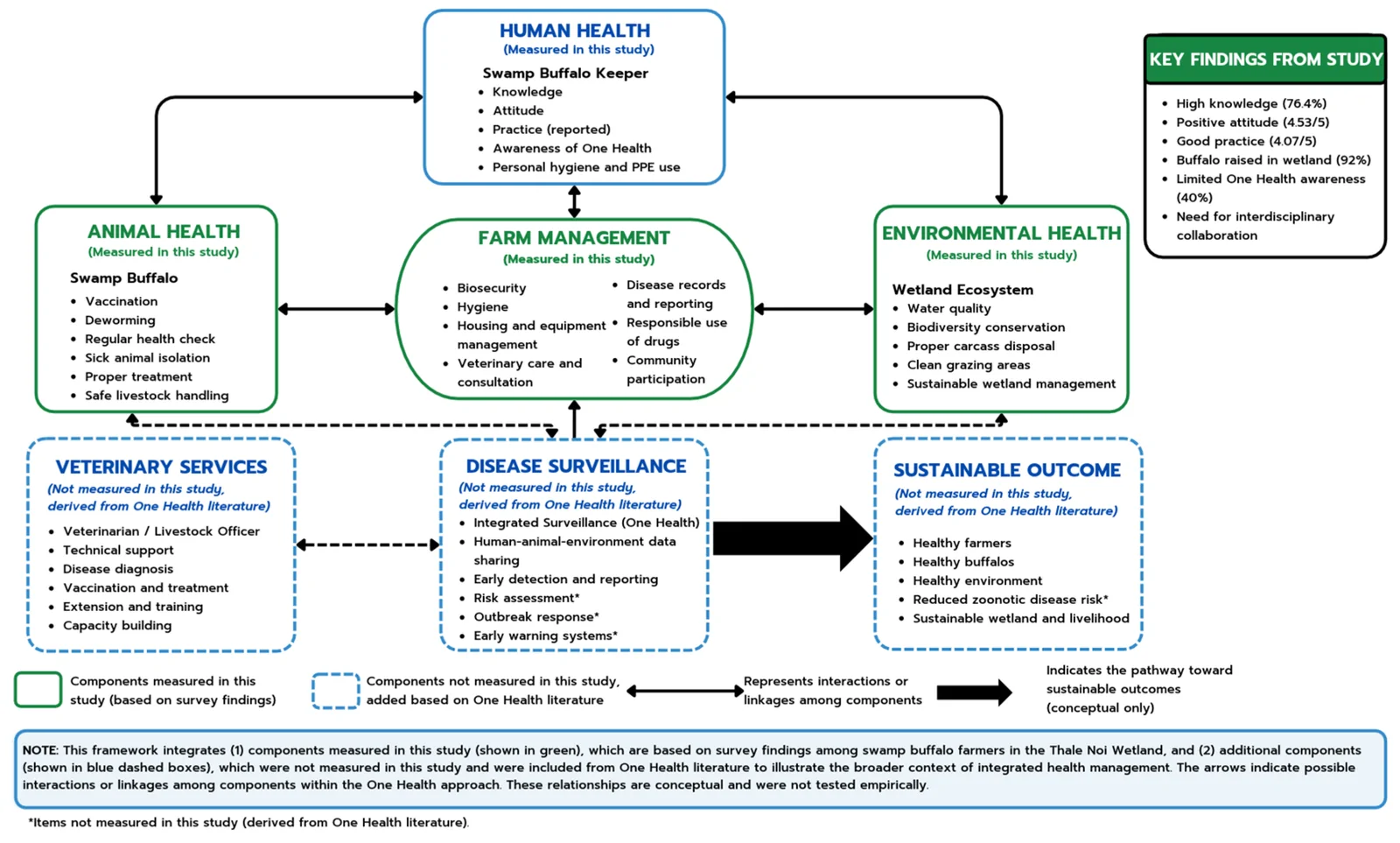
Proposed One Health conceptual framework for swamp buffalo health management in the Thale Noi Wetland, Thailand. Green boxes indicate components measured in the present study and derived from survey findings, whereas blue dashed boxes indicate additional components derived from the One Health literature that were not directly measured in this study. Arrows represent potential interactions or linkages among components and do not indicate tested causal relationships.

Based on the findings of this study, a One Health conceptual framework was developed to integrate the study findings with relevant concepts from the One Health literature and to illustrate potential linkages among participating swamp buffalo keepers’ health, buffalo health, environmental health, farm management, veterinary services, and integrated disease surveillance within the Thale Noi Wetland (Figure 3). The framework is conceptual rather than an empirically validated model, and the arrows represent potential interactions or linkages among components rather than tested causal relationships. The present study directly supports participating swamp buffalo keepers’ health, buffalo health, environmental health, and farm management. Participating swamp buffalo keepers demonstrated relatively high levels of knowledge (76.4%), highly positive attitudes (4.53/5), and good preventive practices (4.07/5). However, only 40% of respondents correctly understood the One Health concept, while 92% reported that buffaloes grazed in the wetland. These findings provide the empirical basis for highlighting the importance of human, animal, and environmental health within the local buffalo production context. Participating swamp buffalo keepers’ management is positioned at the center of the framework as an organizing component that encompasses practices identified in the survey, including biosecurity, hygiene, housing and equipment management, veterinary consultation, disease recording and reporting, responsible use of veterinary drugs, and community participation. This central position is intended to visually represent the potential linkages among the three health domains rather than to imply that farm management has a demonstrated causal effect on them.

Other components shown in Figure 3, particularly veterinary service, integrated disease surveillance, early detection and reporting, environmental monitoring, early warning systems, and sustainable outcomes, were not directly measured in the present survey. We incorporated these components as complementary elements, based on established One Health literature, to provide a broader conceptual context for integrated health management [2,3]. Accordingly, the framework should not be interpreted as evidence that this study empirically demonstrated these components or their relationships. The proposed framework further emphasizes the potential importance of coordination among farmers, veterinary and livestock authorities in addressing health issues at the human–animal–environment interface. This conceptual linkage is consistent with contemporary One Health frameworks, which emphasize collaboration and information sharing across sectors as important elements of integrated disease prevention and surveillance [21,22]. In the context of the Thale Noi Wetland, the framework therefore provides a conceptual basis for considering how survey-identified characteristics and practices may be incorporated into broader One Health activities, rather than constituting a formal assessment of surveillance effectiveness or disease risk.

Overall, the proposed framework integrated empirical findings from the present study with additional concepts derived from the One Health literature. It is intended to serve as a conceptual guide for future research, surveillance planning, and community-based One Health interventions in swamp buffalo production systems, while recognizing that the proposed linkage requires empirical validation in future studies.

### 4.6. Limitations of the Study

This study has several limitations to consider when interpreting the findings. First, the study employed purposive sampling and included only 25 participating swamp buffalo keepers from a target population of 122 swamp buffalo keepers. The researchers did not perform a formal sample size calculation before data collection because the study was exploratory rather than a population-based survey intended to generate statistically representative estimates. For comparison, a conventional sample size calculation based on a population of 122 swamp buffalo keepers, a 95% confidence level, an expected proportion of 50%, and a 10% margin of error would require approximately 55 swamp buffalo keepers. The final sample of 25 participants was therefore smaller than this conventional estimate, limiting the statistical representativeness of the findings. Second, the purposive sampling approach and voluntary participation may have introduced selection bias. Although 62 swamp buffalo keepers were initially approached and invited to participate, only 25 agreed to participate and completed the questionnaire. Participating swamp buffalo keepers may have differed from those who declined in farming practices, knowledge, attitudes, or willingness to engage with researchers. In particular, participants may have been more willing to cooperate or more actively engaged in buffalo farming, which could have influenced the observed findings. Therefore, the results should be interpreted as preliminary exploratory evidence and should not be generalizable to the broader population of participating swamp buffalo keepers in the study area. Future studies should include a larger sample and, where feasible, probability-based sampling to improve representativeness and enable more robust population-level inference.

## 5. Conclusions

This study demonstrated that participating swamp buffalo keepers in the Thale Noi Wetland possess relatively high levels of knowledge, positive attitudes, and good preventive practices regarding buffalo health and zoonotic disease prevention. However, awareness of the broader One Health concept remains limited, indicating a gap between practical husbandry experience and understanding of the interconnected relationships among human, animal, and environmental health. The findings further revealed that the traditional wetland-based buffalo production system creates close interactions among participating swamp buffalo keepers, buffaloes, wildlife, and the surrounding ecosystem, highlighting the importance of integrated health management under the One Health framework. Based on these findings, this study proposes a context-specific One Health conceptual framework that links participating swamp buffalo keepers’ health, buffalo health, environmental health, farm management, veterinary services, and disease surveillance to support sustainable wetland livestock production. Strengthening One Health literacy, improving community-based disease surveillance, and enhancing collaboration among veterinary, public health, and environmental sectors will be essential to reduce health risks and promote the long-term sustainability of swamp buffalo farming and wetland ecosystem conservation in Thale Noi.

## Figures and Tables

**Table 1:** Socio-demographic characteristics of participants in the exploratory study of participating swamp buffalo keepers in Thale Noi Wetland.

Variable	Category	n	%
Gender	Male	17	68.0
	Female	8	32.0
Education	Primary school	17	68.0
	Secondary school	5	20.0
	Vocational certificate/diploma	3	12.0
Main occupation	Agriculture	24	96.0
	Trading	1	4.0
Secondary occupation	Yes	3	12.0

Note: Secondary occupations included fish trap construction, tour boat services, and hired transportation services.

**Table 2:** Quantitative characteristics of participating swamp buffalo keepers and their farming practices in Thale Noi Wetland.

Variable	Mean ± SD	Minimum	Maximum
Age (years)	57 ± 12	33	76
Household size (persons)	4 ± 1	2	6
Annual income from buffalo farming (THB/year)	26,780 ± 9411.96	0	40,000
Buffalo farming experience (years)	32 ± 14	6	63
Number of buffaloes per household (head)	31 ± 41	2	200

**Table 3:** Characteristics of swamp buffalo farming systems in Thale Noi Wetland.

Variable	Category	n (%)
Buffalo farming experience	5–10 years	2 (8.0)
	11–20 years	4 (16.0)
	>20 years	19 (76.0)
	Mean ± SD (years)	32 ± 14
Herd size (head)	<10	7 (28.0)
	10–20	5 (20.0)
	21–50	9 (36.0)
	>50	4 (16.0)
Buffalo production system *	Free grazing	12 (48.0)
	Semi-intensive	12 (48.0)
	Other	1 (4.0)
Primary grazing area *	Wetland	23 (92.0)
	Grassland	2 (8.0)

Note: Values are presented as the number of participating swamp buffalo keepers and percentage [n (%)]. * Buffalo production system and primary grazing area were multiple–response questions; therefore, the total percentages may exceed 100%.

**Table 4:** Attitudes of participating swamp buffalo keepers toward integrated health management under the One Health framework.

Attitude Statement	Mean ± SD	Level
Regular health check-ups for buffaloes are essential.	4.72 ± 0.54	Highest
Disease prevention is more important than disease treatment.	4.56 ± 0.65	Highest
Wearing personal protective equipment (PPE) when handling buffaloes should become routine practice.	4.20 ± 0.82	High
Environmental quality directly affects the health of both buffaloes and humans.	4.56 ± 0.71	Highest
Livestock officers or veterinarians should be notified when sick buffaloes are detected.	4.52 ± 0.71	Highest
Veterinary drugs should only be used according to veterinarians’ recommendations.	4.56 ± 0.58	Highest
Community participation is essential for disease surveillance and prevention.	4.58 ± 0.65	Highest
Overall attitude score	4.53 ± 0.66	Highest

Note: Attitude levels were interpreted using a 5-point Likert scale: 4.21–5.00 = Highest, 3.41–4.20 = High, 2.61–3.40 = Moderate, 1.81–2.60 = Low, 1.00–1.80 = Lowest.

**Table 5:** One Health-based disease prevention and buffalo health management practices among participating swamp buffalo keepers.

Practice Statement	Mean ± SD	Level
Wear boots when entering buffalo grazing areas.	3.60 ± 1.32	High
Wash hands after handling buffaloes or animal carcasses.	4.16 ± 0.94	High
Deworm buffaloes according to the recommended schedule.	3.72 ± 1.28	High
Isolate sick buffaloes from the herd.	4.12 ± 1.33	High
Regularly clean buffalo housing and equipment.	4.12 ± 1.13	High
Seek advice from a veterinarian when buffaloes become sick.	4.28 ± 0.74	Highest
Maintain records of buffalo diseases, treatments, and drug use.	3.92 ± 1.50	High
Avoid direct contact with the blood or body fluids of buffaloes.	4.20 ± 1.22	High
Overall practice score	4.07 ± 1.16	High

Note: Practice levels were interpreted using a 5-point Likert scale: 4.21–5.00 = Highest, 3.41–4.20 = High, 2.61–3.40 = Moderate, 1.81–2.60 = Low, 1.00–1.80 = Lowest.

**Table 6:** Potential health risks identified from the study under the One Health framework.

One Health Component	Potential Risk	Evidence from This Study	Potential Impact
Buffalo Health	Exposure to infectious and parasitic diseases due to free grazing	92% of buffaloes grazed in wetlands; 48% of participating swamp buffalo keepers reported free-grazing and 48% semi-intensive systems	Potential opportunities for contact among buffaloes, wildlife, and shared environmental resources
	Inadequate understanding of integrated disease prevention	Only 40% of participating swamp buffalo keepers correctly understood the One Health concept	Potential limitation in understanding interconnected human, animal, and environmental health
	Inconsistent implementation of preventive practices	Overall reported practice score = 4.07 (High); deworming and biosecurity practices were not at the highest level.	Areas for strengthening routine herd health and preventive practices
Participating swamp buffalo keepers’ health	Potential exposure to zoonotic hazards during routine buffalo handling	Participating swamp buffalo keepers frequently handle buffaloes in free-ranging production systems	Potential occupational exposure to zoonotic hazards
	Inconsistent use of personal protective equipment (PPE)	PPE-related practices were less consistent than other preventive practices	Potential occupational health concern and area for improving personal biosecurity
	Limited One Health literacy	Only 40% currently understood the One Health concept	Potential limitation in understanding interconnected health risks among humans, animals, and the environment
Environmental Health	Frequent interaction among buffaloes, wildlife, and wetland ecosystems	92% of buffaloes grazed in the Thale Noi Wetland	Frequent ecological interactions among livestock, wildlife, and aquatic ecosystems
	knowledge gap regarding carcass disposal	76% correctly recognized the importance of proper carcass disposal	Potential concern regarding appropriate environmental sanitation and carcass management
	Degradation of wetland resources	92% of buffaloes in wetland	Potential implications for sustainable wetland management and ecosystem resilience

## Data Availability

The original contributions presented in this study are included in the article. Further inquiries can be directed to the corresponding author.
